# Polypoid heterotopic gastric mucosa: in terminal ileum causing extensive lower gastrointestinal bleeding without Meckel’s diverticulum: a case report

**DOI:** 10.1186/s13256-024-04644-7

**Published:** 2024-08-06

**Authors:** Saeid Aslanabadi, Davoud Badebarin, Nazila Hasanzadeh Ghavifekr, Babollah Ghasemi, Maryam Shoaran, Marjan Hesari

**Affiliations:** 1 Section of Pediatric Surgery, Department of Surgery, Tabriz University of Medical Sciences, Tabriz, Iran; 2 Department of Pathology, Tabriz University of Medical Sciences, Tabriz, Iran; 3 Department of Pediatrics, Tabriz University of Medical Sciences, Tabriz, Iran; 4 Tabriz University of Medical Sciences, Tabriz, Iran

**Keywords:** Heterotopic gastric mucosa, Ileum, Polyp, Gastrointestinal bleeding

## Abstract

**Background:**

Heterotopic gastric mucosa (HGM) can be located in various parts of the gastrointestinal tract. As a rare anomaly in the small intestine, it can become complicated by intussusception, obstruction, gastrointestinal bleeding, and even peritonitis, leading to death.

**Case presentation:**

This case report focuses on a 12-year-old Middle Eastern boy who presented with hematochezia and abdominal pain for a couple of days. A tagged Red blood cell (RBC) scan and Technetium scan revealed gastrointestinal bleeding at the lower abdomen, highly suggestive of the diagnosis of Meckel’s diverticulum. Subsequently, exploratory laparotomy revealed contiguous and scattered mucosal lesions with multiple polyps of various sizes in the terminal ileum. Meckel’s diverticulum was absent, and the patient was treated with resection and primary anastomosis. The resected tissue revealed extensive ectopic gastric mucosa and polypoid tissues. The patient recovered uneventfully and was discharged four days after the surgery. The symptoms did not recur within six months after his surgery.

**Conclusion:**

Our case demonstrated that despite the rarity of multiple polypoid gastric heterotopias in the terminal ileum, it should be considered as one of the differential diagnoses of gastrointestinal tract bleeding.

## Background

Heterotopic gastric mucosa (HGM) has been defined as gastric tissue with its specific histological characteristics, localizing outside of the stomach [1]. The healing process of damaged mucosa can lead to gastric metaplasia, and it is crucial to differentiate them from heterotopia. True heterotopia is a congenital developmental lesion that can mostly be found anywhere in the alimentary tract from the tongue to the rectum [2–4]. It is more common within the Meckel’s diverticulum and gastrointestinal duplications [5]. The reported incidence of HGM in the esophagus is relatively high, ranging from 0.1 to 13.8% [6]. Duodenum is also a common site of involvement, but overall involvement of the small intestine is an uncommon condition [5, 7].

Literature suggests that the occurrence of HGM as multiple polypoid gastric tissues in the small intestine, particularly in the ileum [7], and the presentation with gastrointestinal bleeding (GIB) is quite rare, and probable diagnosis requires high clinical suspicion. Moreover, the definite diagnosis is often made postoperatively by the study of the histopathologic specimen [8, 9].

We present a case of HGM complicated by massive GIB. To the best of our knowledge, this is an extremely rare case characterized by polypoid gastric heterotopias in the small intestine and was treated successfully by surgical resection.

## Case presentation

A 12-year-old Middle Eastern boy was admitted to the pediatric surgery ward with an episode of hematochezia and mild abdominal pain. The hematochezia started two days prior to the patient’s admission with massive painless rectal bleeding. Additionally, there was an instance of melena occurring once to thrice daily.

The patient was a normal-appearing healthy boy with morphological status proportional to his age. The only past medical history consisted of hypogammaglobinemia and neutropenia at the age of six treated by intravenous immunoglobulin infusion. Family history was also negative for gastrointestinal disorders.

Minor intermittent abdominal symptoms as periumbilical pain was present without fever or vomiting.

On the physical examination, the patient was mildly ill-appearing with pale conjunctiva. He represented a mild tenderness in the periumbilical area without any rebound tenderness or abdominal distention. Digital rectal examination revealed black, tarry feces consistent with melena. All other examination findings were within normal limits. No additional abnormalities were found during the physical examination.

The patient was initially treated with administration of intravenous fluids to replete fluid loss due to GIB. Complete blood count and coagulation tests were taken, and a packed cell was reserved. The patient continued to have stable hemodynamics, whereas considerable hematochezia was repeated twice on the first day of admission. Lab study upon admission revealed mild anemia (Hb level = 11.5 g/dL, Hct = 33.1%, MCV = 87 fL, WBC count = 3.6 × 10^3^/μL per microliter) as well as a positive stool guaiac test in three consecutive samples. Other lab values, including biochemical profiles and coagulation studies, were within normal limits.

**Fig. 1:**
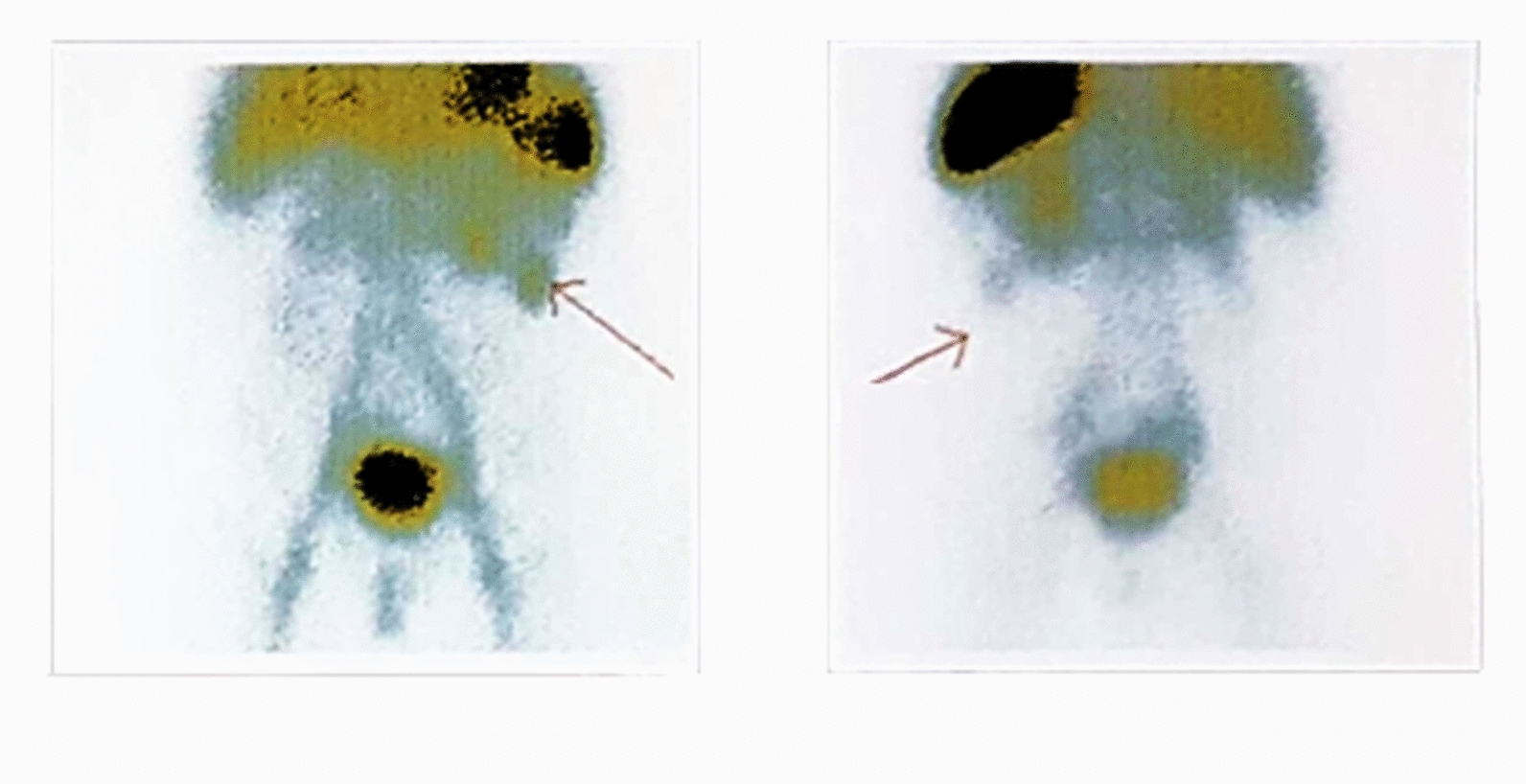
Arrow indicates abnormal activity accumulation (Tagged RBS scan with 99m pertechnetate) in the Left lower quadrant at the 90th min after injection, suggestive of intestinal bleeding

**Fig. 2:**
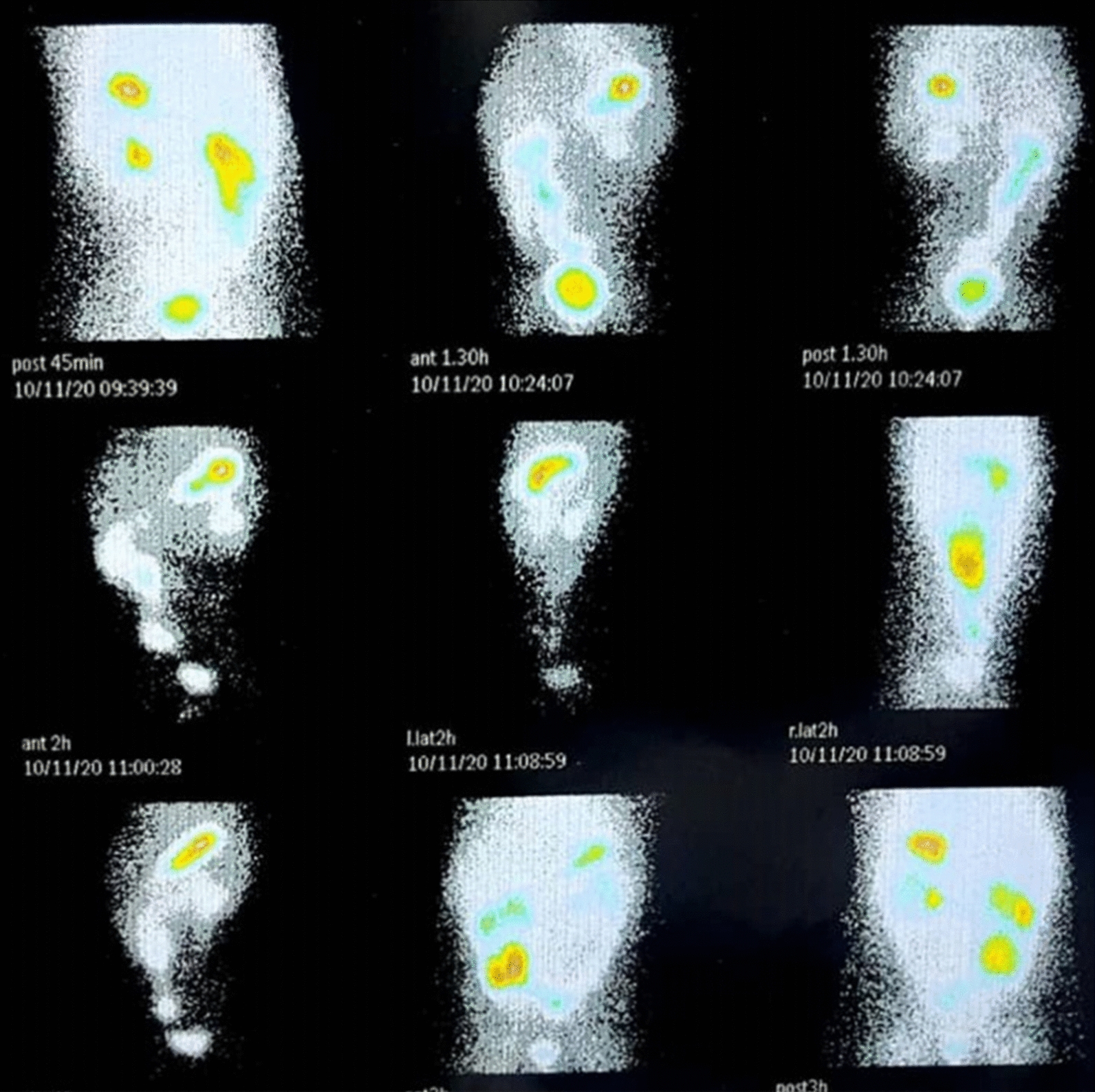
Technetium-99m pertechnetate radionuclide study (Meckel scan) demonstrating Longitudinal activity accumulation in the right peri-umbilical suggesting Meckel’s diverticulum

An abdominal ultrasonographic (US) study was performed, which revealed uncharacteristic findings and showed no intra-abdominal abnormalities. To further assess the patient presenting with signs of GIB, a red blood cell (RBC) scan was performed, suggesting GIB at the LLQ (Fig. 1). Considering the patient’s age and ethnicity profiles, the result of the RBC scan was highly suggestive of bleeding from a Meckel’s diverticulum. Despite the normal US findings, due to the RBC scan result and the physician’s highest suspicion of Meckel’s diverticulum, a Meckel scan with 99mTc-pertechnetate was performed. The scan revealed a heterogeneous enhancement in the subhepatic region during the dynamic phase that appeared simultaneously with gastric activity. Although not in a typical appearance, it was suggestive of Meckel’s diverticulum (Fig. 2).

Based on these findings, the patient was prepared for laparotomy exploration with the most probable diagnosis of Meckel’s diverticulum. Under general anesthesia, laparotomy was performed with a transverse right upper quadrant approach. Upon initial inspection, the peritoneal cavity was intact, with no blood or free fluid accumulation inside the peritoneal cavity. On assessing the ileum, a segment of the terminal ileum, approximately 15–20 cm in length and situated about 20 cm proximal to the ileocecal junction, was observed to be mildly dilated (5–40 mm) and dysmorphic. Multiple palpable polyps were palpable in the affected area. The enterotomy was done, and morphological appearance revealed severe mucosal thickness with altered and deep mucosal folds and polypoid multiple lesions varied in size. The involved segment of the ileum was resected with about 1–2 cm of grossly normal bowel. The pathology showed 6 mm and 12 mm safe margins on proximal and distal ends. Ileileostomy was performed by establishing end-to-end anastomosis of both ends of the ileum.

**Fig. 3:**
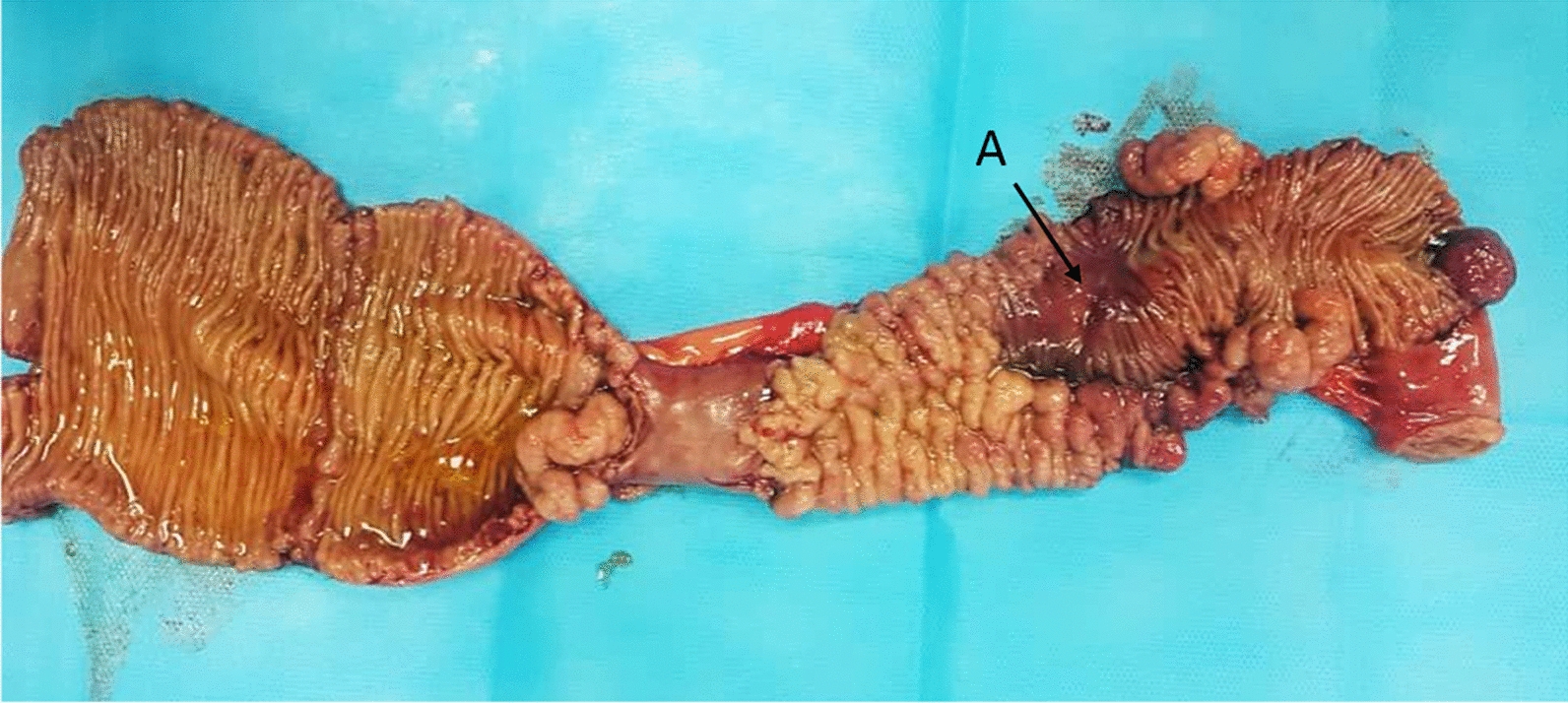
Resected segment of ileum; Dilated lumen with various patterns of mucosal rearrangement and multiple polyps with one obviously ulcerated polyp (**A**)

As depicted in Fig. 3, a section approximately 30–35 cm in length was resected from the ileum. On gross examination, the resected portion contained multiple polyps with different sizes and shapes measuring from 5 × 5 × 10 to 40 × 25 × 20 mm in diameter for the smallest and largest ones, respectively. There was no sign of obstruction, It only appeared that one of the polyps had caused spatial restriction in the lumen.

**Fig. 4:**
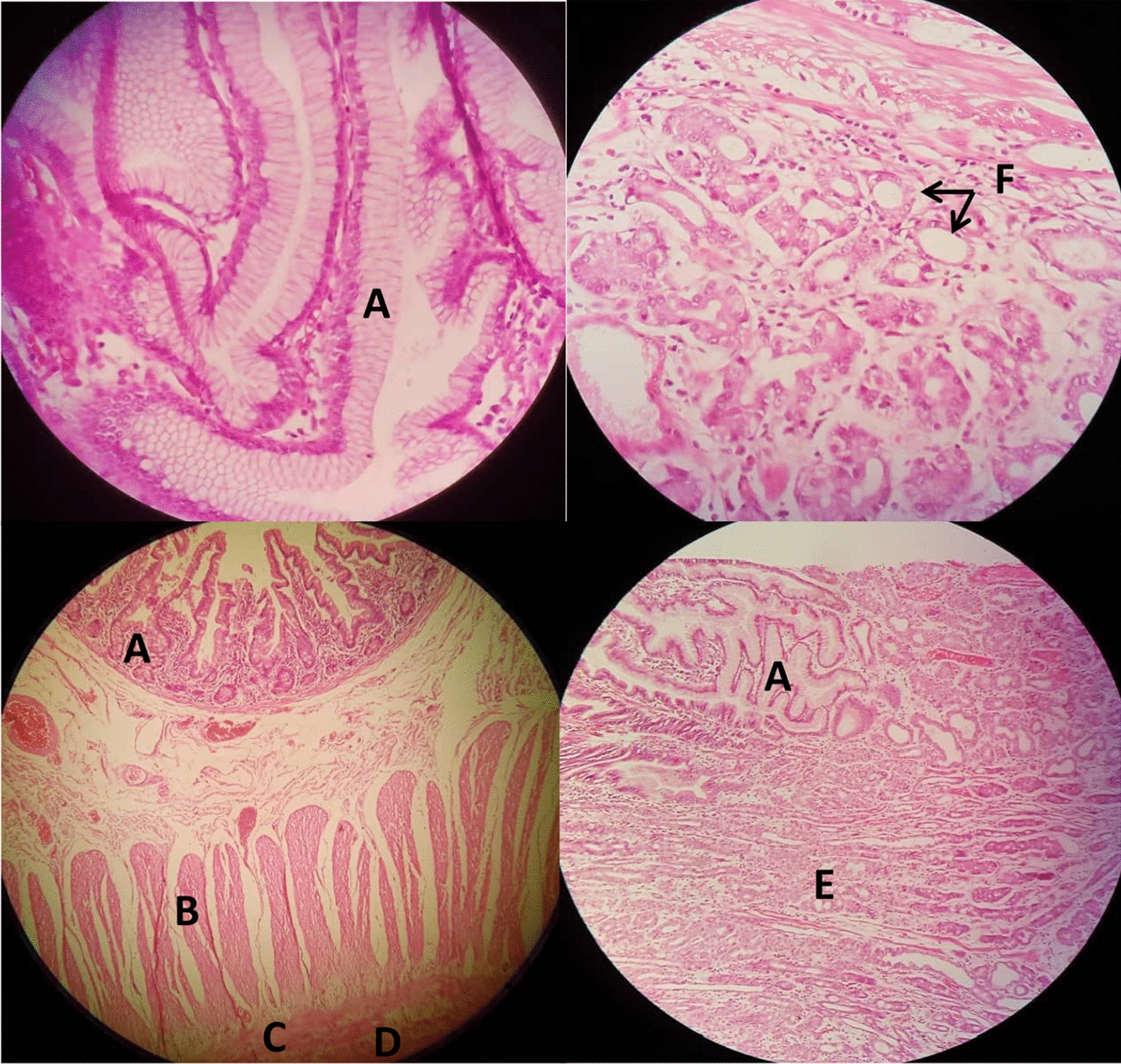
Histopathological sections of specimen within HGM polyps; (**A**) Faveolar epithelium of gastric mucosa, (**B**) Inner muscular layer, (**C**) outer muscular layer, (**D**) Nerve bundles and G. cells, (**E**) Gastric crypts, (**F**) Gastric glands

Microscopic and histopathologic examination (Fig. 4) of the specimen revealed multiple polypoid tissues with intact surface epithelium and focally engorged and congested blood vessels as well as ectopic gastric type glands within all polyps. The surface epithelium of polyps was villous shaped, consisting of foveolar, secretory, and intestinal villi epitheliums. No dysplastic changes were observed in the evaluated tissue specimen.

The recovery and post-op courses were uneventful. Oral nutrition was initiated on the second postoperative day, and the patient was discharged on post-op day four (POD-4) with short-course (5-day) oral antibiotic therapy with a combination of cefixime and metronidazole.

The patient was symptom-free and healthy in multiple follow-up examinations within six months after his surgery. There is no sign of the recurrence of the patient’s symptoms to date.

## Discussion

Multiple polypoid gastric heterotopias is a rare congenital disorder of the gastrointestinal tract, especially in the small intestine. Although this condition can occur all over the gastrointestinal tract [2], it is more common with congenital anomalies such as Meckel’s diverticulum [5]. In the anatomically normal gastrointestinal tract, HGM is common in the upper esophageal sphincter [10], with a prevalence of approximately three percent [11]. Duodenum also represented HGM with a prevalence of around 0.5% to 8.9%, according to different reports [11]. HGM is extremely rare in other regions. The clinical presentation of HGM can vary significantly and may comprise a broad spectrum of clinical symptoms case-wise. Many patients remain asymptomatic, though others may experience severe complications, including gastrointestinal bleeding, perforation, and obstruction [4, 12, 13]. Our case was unique in terms of the location and appearance of lesions, which appeared as multiple polyps composed of heterotopic mucosa in the small intestine.

The pathogenesis of HGM is not thoroughly understood. However, some theories have been proposed to elucidate the origin of HGM. The theory of acquired HGM results in a regenerative process due to the inflammatory breakdown of the normal intestinal mucosa is not supported anymore [5]. The probable hypothesis is that HGM is a result of abnormal differentiation of pluripotent stem cells with the ability to differentiate into multiple epithelial cell types; this theory has been widely accepted [5, 14].

As the HGM could remain asymptomatic even for a lifelong, the true incidence of this lesion is quite unclear. The most common complications in the small intestine are intussusception, bowel obstruction, ulceration with or without perforation, and bleeding [4, 5, 12, 15]. Clinical presentation and complications are widely varied and depend on the size, location, and tissue pattern of HGM [5]. In our case, the symptoms were acute, and the bleeding was the dominant presentation.

According to Taylor (1927), the result from the autopsy of 150 patients demonstrates the incidence of heterotopic gastric mucosa about 1.5%. Except for life-threatening surgical emergencies that require prompt surgical intervention, evaluation of symptomatic patients using a diversity of imaging modalities could be beneficial to confirm the diagnosis of HGM. Among them, modalities such as X-ray, US, CT scan, and angiography are less accurate for detecting HGM [16]. As in our case, ultrasonography was inconclusive.

A tagged red blood cell scan with 99mTc-pertechnetate is a useful modality in evaluating GIB, but the specificity for HGM is low with the poor localizing ability. In our case, it also contains limited diagnostic information.99mTc-pertechnetate radionuclide study (Meckel scans) is an accurate, non-invasive imaging modality that can be used to detect gastrointestinal bleeding in HGM. There are different kinds of scintigraphy patterns for patients who underwent tech 99. This depends on the location, size of the heterotopic mucosa, and the amount of active bleeding [7]. The sensitivity of scintigraphy in detecting HGM is high. However, according to Rosenthal *et al. *(1972), the incidence of false negatives in scintigraphy is reported at about 50%. Nonetheless, the exact distinction between HGM and Meckel’s diverticulum containing gastric tissue is impossible due to the same tissue intake. Moreover, the specificity of this modality is low in differentiating Meckel’s diverticulum from ectopic HGM. As described above, a 99mTc scan was performed for our patient and was highly suggestive of Meckel’s diverticulum with severe bleeding, which is the most common diagnosis.

In summary, the definite diagnosis of HGM is usually made during exploratory surgery and confirmed by a post-operative histological evaluation of the resected tissue specimen [10, 14]. Patients with HGM may be amenable to surgical intervention. However, a decision for surgical management must be taken according to the patient’s presentation, hemodynamic status, and probable complications due to the underlying disorder. We describe a rare case of multiple polypoid gastric heterotopias in the terminal ileum. Explorative laparoscopic surgery with histopathologic examination was the definitive method in clinical diagnosis [9]. The progression or susceptibility to malignancy associated with this lesion is uncertain. However, due to the limited involvement, surgical resection is the preferred therapy. Although heterotopic gastric mucosa is one of the rare causes of GIB, it should be considered as a differential diagnosis of painless lower GIB.

## Conclusion

This case showed the need for clinical suspicion and thorough evaluation, including imaging studies, in managing rare gastrointestinal anomalies such as multiple polypoid gastric heterotopias. Despite heterotopic gastric mucosa being one of the rare causes of bleeding, it should be considered in a differential diagnosis of alimentary tract bleeding.

## Data Availability

All datasets of this study are available from the corresponding author on reasonable requests.

## Abbreviations

HGM: Heterotopic gastric mucosa
GIB: Heterotopic gastric mucosa
US: Ultra-sonographic
RBC: Red blood cell
GIB: Gastrointestinal bleeding
